# Social Validity in Spain of the *Mantente REAL* Prevention Program for Early Adolescents: Social Validity of Mantente Real in Spain

**DOI:** 10.1007/s10935-022-00701-3

**Published:** 2022-08-04

**Authors:** Olalla Cutrín, I. Mac Fadden, F. F. Marsiglia, S. S. Kulis

**Affiliations:** 1 Department of Psychology and Psychobiology, Universidade de Santiago de Compostela, Calle Xosé María Suárez Núñez, s/n, 15782 A Coruña, Santiago de Compostela, Spain; 2 Department of Sociology, Universidad Pablo de Olavide, Sevilla, Spain; 3 Global Center for Applied Health Research, Arizona State University, Phoenix, AZ United States; 4 School of Social Work, Arizona State University, Phoenix, AZ United States; 5 School of Social and Family Dynamics, Arizona State University, Tempe, AZ United States

**Keywords:** Substance use, Adolescence, Resistance strategies, Prevention program, Social validity, Spain

## Abstract

Studies focusing on the relevance or impact of a program, not just on its efficacy or effectiveness, can make important contributions to prevention science. This article documents the social validity (i.e., a construct encompassing feasibility, acceptability, and utility) of a universal substance use prevention program for early adolescents in Spain. The *Mantente REAL* (*keepin’it REAL*) program was culturally adapted to the Spanish context, implemented, and evaluated in six public middle schools in two regions of Spain. Participating teachers (*N* = 15), students (*N* = 354), and research team members (*N* = 6) reported on the feasibility, acceptability, and utility of the program implemented with first grade secondary school students. Qualitative and quantitative data about the program, its curriculum, and the implementation process were collected through teachers’ focus groups, students’ surveys, and observation forms completed by members of the research team. *Mantente REAL* was perceived to be a prevention program that was feasible for implementation in Spanish middle schools, although some logistics related to school structural constraints should be addressed in future implementations. The topics and activities in the curriculum were highly accepted by teachers and students, and they reported that the program was useful in teaching resistance strategies to cope with substance use and other risky situations. The findings support the social validity of the culturally adapted *Mantente REAL* program for early adolescents in Spain, and highlight how feedback from stakeholders involved in the implementation can improve the dissemination of effective prevention approaches.

The aim of designing and evaluating evidence-based prevention programs is to assure that youth have access to quality, efficient and effective interventions that can prevent or delay their use of alcohol and other drugs and increase the wellbeing and overall health of communities (European Monitoring Centre for Drugs and Drug Addiction, EMCDDA, 2011; National Institute on Drug Abuse, 2021). The high substance use rates among European youth, and more specifically Spanish youth, highlight the importance of implementing science-based and efficacious prevention programs. The National EDADES 2019/2020 Study reports high rates of drug use and abuse in Spain among adolescents. For example, 15.4% of all respondents reported binge drinking in the last 30 days, 63% of them reported drinking alcohol, and one out of three adolescents reported getting drunk in the last 30 days (Observatorio Español de las Drogas y las Adicciones, 2021).

The EMCDDA (2011) established quality standards to guarantee effective and efficacious drug abuse prevention. The principles encompass the entire prevention process, from assessment of needs and resources to the final evaluation and dissemination of efficacious interventions (Barth et al., 2012). Disseminating culturally adapted programs, rigorously evaluated in a controlled setting and with demonstrated effectiveness, increases the probability of congruence between the key components of a program, its expected outcomes and the probabilities that programs will be adopted and implemented (Barrera et al., 2017). The implementation of those standards is, however, uneven across European Union member countries (EMCDDA, 2021).

In Spain, 30 school prevention programs are listed in the evidence-based prevention database (Sociedad Científica Española de Estudios sobre el Alcohol, el Alcoholismo y las otras Toxicomanías, 2021). The evidence considered by the database focuses on efficacy. However, previous research has indicated that universal school-based drug prevention interventions usually show low levels of efficacy (Espada et al., 2015; Tanner-Smith et al., 2018). Therefore, other indicators, such as social validity, should be considered to evaluate universal prevention programs. An evidence-based intervention identified as efficacious will have a greater prevention impact if it is perceived by the end users as acceptable, appropriate, feasible and sustainable (Murta et al., 2021). These constructs have been commonly used as indicators of implementation outcomes that help measure implementation success and are key intermediate outcomes that facilitate intervention effectiveness (Proctor et al., 2011). Moreover, information about the process of implementation and the adequacy of the curriculum is essential not only for a successful implementation, but for long-term sustainability (Harthun et al., 2009; Soneson et al., 2020). To advance knowledge on these issues, the field of prevention is focusing on the social validity, relevance or impact of the program, in addition to its efficacy or effectiveness.

The conceptual taxonomy of Proctor et al., (2011) provides a theoretical background for implementation research and a guide to a methodological approach, addressing a shortcoming in conceptual clarity based on the psychometric properties of measures. Previous models indicated the relevance of three variables in the assessment of implementation outcomes: feasibility, acceptability, and appropriateness (Weiner et al., 2017). The latter two are highly overlapping concepts and recent research assessing the actual functioning of prevention programs in real settings points to the impact of the program as a variable to be considered as an outcome indicator of implementation (Murta et al., 2021).

According to Murta et al., (2021) “social validity studies are meant to analyze the social relevance of public health intervention goals, the appropriateness of their strategies, and the social significance of their impact, as perceived by the target audience” (p. 660). From a social validity perspective, a dynamic and cyclical process for assessing implementation allows intervention research to redefine and optimize programs and, subsequently, augment the effectiveness of the intervention in real settings and specific contexts. In this process, the information provided by both the participants and the facilitators of the intervention is useful to evaluate the significance of the program (Murta et al., 2021).

Considering the previous theoretical, methodological, and social models, as well as cultural responsiveness, this study investigated social validity by assessing whether a particular prevention program is (1) feasible to be implemented (in the specific context with the target population and the available resources), (2) acceptable regarding contents and activities, and understandable, and/or (3) useful to gain knowledge and impact participants’ lives.

## ***Keepin’ it REAL*in Spain**: ***Mantente REAL***

*keepin’ it REAL*, a program originally designed for a multicultural population of middle school students in the US, was demonstrated efficacious in multiple studies in the United States (Hecht et al., 2003; Kulis et al., 2007; Marsiglia et al., 2011). The original version of *keepin’ it REAL* (Marsiglia & Hecht, 2005), designed to be delivered by regular classroom teachers in middle schools, has been culturally adapted internationally and has shown to be effective in different social and cultural contexts, including in Mexico, Guatemala, and Uruguay (Kulis et al., 2019; Kulis, Garcia-Pérez et al., 2021; Kulis, Marsiglia et al., 2021; Marsiglia et al., 2018). A recent trial of the more recent DARE version of *keepin’ it REAL* in Brazil reported more mixed evidence, but this version was delivered by police officers to elementary school students younger than the target population of the original version (Valente & Sanchez, 2022).

The intervention teaches life skills and four specific strategies to manage different risky situations, summarized in the acronym of REAL: Refuse by saying no verbally or non-verbally; Explain why you don’t want to use substances; Avoid situations where substances are consumed or available; and Leave situations where substances are being used. Participants learn the REAL strategies as a tool of empowerment and other life skills to assess risks associated with substance use and to make decisions about it. The intervention validates the youth’s beliefs and attitudes that contribute to their positive development. Because *keepin’ it REAL* is designed to integrate the sociocultural values ​​of the participating adolescents, the manuals and accompanying videos have been culturally adapted when the program has been implemented in countries outside the United States (Marsiglia et al., 2019).

*Mantente REAL* is the Spanish language version of this universal prevention program for secondary school adolescents, consisting of 12 sessions and 5 videos produced in each home country. Although the collaboration between a binational Spanish-US team dates back to 2005 (Luengo et al., 2008), *keepin’ it REAL* was culturally adapted in Spain between 2015 and 2019 through three main phases by funded studies. First, a pilot study in Seville was conducted (2015–2017) to test the feasibility and acceptability of the Mexico-based *Mantente REAL* version in Spain (Marsiglia et al., 2019). This version was selected to be implemented in Spain because it was the most recently produced Spanish language version of the *Mantente REAL* curriculum. This pilot also aimed to test the potential effect sizes of the program for the prevention of drug use among adolescents in the Spanish context and to identify the socio-cultural adaptation needs. One of the main findings of the study pointed to the need to culturally adapt the videos and the activities (e.g., the examples provided, the characters presented, and the type of Spanish vernacular used).

Phase two consisted of a cultural adaptation study conducted in Seville (2017–2018). Students and teachers from two middle public schools in Seville participated in the adaptation process. The study focused on the adaptation of the materials of the program (2 manuals and 5 videos) to the Spanish context. The adaptation of the content of the videos involved 32 students between 11 and 12 years old participating in four focus groups, who worked on script development for the five videos. More than 60 students from the same two schools participated in producing the videos, with the support and technical assistance of a professional videographer. Based on the feedback received from teachers, students and observers, researchers from the binational team and 5 external experts revised the manuals. Once the culturally adapted curriculum was finished and revised, a new study was conducted to assess its efficacy.

Phase three consisted of a small Randomized Control Trial (RCT) study (2018–2019) to test the efficacy of the adapted version of the program in the two sites involved in the adaptation process, Seville and Santiago de Compostela. The study was conducted in 12 public secondary schools of medium socio-economic level (6 from each site). The total sample included 755 first grade secondary school students (equivalent to 7th grade in the US). The results suggest the program was efficacious in curbing the increase in excessive alcohol consumption and intoxication episodes among students who received the *Mantente REAL* intervention, relative to treatment-as-usual controls (Cutrín et al., 2020).

## The current study

After finding preliminary evidence of its efficacy in the small RCT study, the present study assessed the social validity of *Mantente REAL* in the Spanish context, examining factors that contribute to the quality, impact and sustainability of this evidence-based and culturally adapted prevention intervention. This study aimed to analyze the feasibility, acceptability, and utility of the *Mantente REAL* program in the first grade of Secondary Compulsory Education in Spain, equivalent to 7th grade in the United States. Because *Mantente REAL* was developed by youth and for youth, we hypothesized, from a social validity perspective, that the culturally adapted version of the intervention for Spain would be evaluated as feasible, acceptable and useful by the participants and facilitators. To provide the perspectives of different informants involved in the implementation of *Mantente REAL*, from the two different regions, we analyzed data evaluating the intervention from the perspective of participating students, teachers (i.e., program facilitators), and the research teams’ observations of fidelity during the implementation process.

## Method

### Participants

The sample of this study came from the small cluster randomized trial testing the efficacy of the culturally adapted *Mantente REAL* in Spain. The 12 selected state schools from Seville and Santiago de Compostela were randomized into two conditions: *Mantente REAL* (schools implementing the program) or control (treatment-as-usual schools). Schools in both sites, Seville and Santiago, were assigned to control and intervention conditions (3 schools per site and per condition). All students enrolled in the first grade of the selected schools were invited to participate and 80.1% of that target population participated in the study (see Cutrín et al., 2020, for sampling and implementation details). For the present study, the analysis is limited to the *Mantente REAL* condition, considering only students attending schools assigned to the experimental group (*N* = 354; 6 schools) and teachers implementing the program (*N* = 15), as well as members of the field research teams serving as observers (*N* = 6).

Participating students were enrolled in the initial grade of secondary school (47.3% female; aged 11 to 15, *M*_age_ = 12.3; *SD* = 0.59). The teachers implementing *Mantente REAL* were 53.3% female, aged 24 to 50 (*M*_age_ = 38.64; *SD* = 7.41), and had an average of 12 years teaching experience. Members on the research teams from the two participating universities included the field coordinators of the project and graduate students who performed roles as liaisons to the schools and observers of implementation fidelity.

### Procedure

#### Description of the intervention

In the schools assigned to the intervention group, the program was implemented by the regular tutors (homeroom teachers) in their classrooms during school hours. Before starting the curriculum’s lessons, all 1st grade (7th grade in the US) tutors received practical training over a two-day long session to learn how to deliver the *Mantente REAL* curriculum with fidelity. Program implementation was structured and guided by a Teacher’s Manual and a Student’s Workbook, which detail all the procedures for teachers to follow and the activities to be completed by students. *Mantente REAL* has a total of 12 lessons and teachers were generally able to deliver one lesson per week. Teachers began delivery of the curriculum lessons in January of 2019 and completed its implementation in April of the same year.

#### Data collection procedures

Ethical research review committees for the Spain and US research teams approved the study’s research design and all qualitative and quantitative data collection procedures. Information about feasibility, acceptability, and utility was collected through focus groups with teachers, students’ surveys, and the research team’s observations of fidelity during the implementation process.

**Teachers focus groups.** All teachers (*N* = 15) from the six schools that implemented *Mantente REAL* were invited to participate in focus groups about a month after finishing the program implementation. All of them accepted the invitation and gave their assent to participate in about a one-hour focus group. In each site, focus groups were conducted with teachers in each school (6 focus groups; around 2–4 teachers per school) to get information about the implementation process and opinions about the *Mantente REAL* curriculum. All the focus groups were recorded and transcribed in Spanish (in Santiago de Compostela some of the teachers used the Galician language, but their comments were transcribed into Spanish).

**Student post-test surveys.** Students (*N* = 354) enrolled in the initial grade level of the six secondary schools assigned to the intervention condition completed a post-test survey about a month after finishing the program implementation. Parents provided informed consent for their child to participate in the survey, and students gave their assent before completing the survey. Qualified members of the research teams informed students that completing the survey was voluntary and answers would remain confidential, and provided proper instructions to answer the self-reported survey in a 50-minute classroom session. Questions assessing different aspects of *Mantente REAL* and students’ opinions about the program were included at the end of the post-test survey.

**Fidelity observations.** Qualified members of the research teams visited the classrooms of each implementing teacher at sessions 1, 3, and 7 to rate fidelity to the manual, classroom management, and student participation. The observers completed standardized forms with closed-ended ratings of various aspects of fidelity and participants’ involvement, and open-ended comments about implementation issues and feedback provided to teachers.

### Measurements

#### Feasibility

The degree to which the program was viable to be implemented was explored through the following measures that directly affect program implementation (Bird et al., 2014; Lohan et al., 2018; Soneson et al., 2020).

**Qualitative measures from teachers’ focus groups.** (1) *Intervention fit*: perception of the program as relevant to address the prioritized goals (substance use) and appropriate to the target population (1st grade secondary school students). (2) *Implementation characteristics*: complexity of the delivery of the curriculum, helpfulness of the manual, and time adequacy (or time concerns) to implement the program. (3) *Practicality*: engagement in delivery by teachers and participation by students.

**Quantitative measures from students’ post-test surveys.** 1) *Practicality*: students reported on the post-test survey how many videos they watched (0–5) and how much they participated in the program (0 = not at all, 1 = a little, 2 = some, 3 = a lot).

**Quantitative measures from researchers’ observation forms.** (1) *Practicality*: observers rated three closed-ended items to assess student involvement with the curriculum (i.e., participation, engagement, and attention to the videos). (2) *Fidelity*: observers informed about fidelity using multiple measures. One was whether the teacher completed each of 8 or 10 curriculum items that were specific to the observed lesson (e.g., introduced a particular topic, completed an activity in the student manual). Two of the three sessions observed have an 8-item curriculum checklist; the other session has a 10-item checklist. These were combined into an index counting the number completed, from 0 to 10. Additionally, single items assessed how well the teacher was prepared for the lesson, informed about its content, followed the lesson plan, gave clear instructions, and motivated students to participate (1 = not at all, 2 = somewhat, 3 = mostly, 4 = completely). Another two items rated the teacher’s class management of the group processes in the curriculum (1 = poor, 2 = well, 3 = excellent) and whether the pace of instruction was appropriate (1 = too fast, 2 = too slow, 3 = appropriate). One more item assessed whether the teacher added content not included in the manual (0 = no, 1 = yes).

#### Acceptability

The degree to which the participants accepted the program was explored through the following measures (Lohan et al., 2018) that assess the perceived appropriateness, fairness, and reasonableness of an intervention for addressing an identified need (Gadke et al., 2021; Nastasi & Truscott, 2000).

**Qualitative measures from teachers’ focus groups.** (1) *Satisfaction*: overall satisfaction with the program (e.g., enjoyed implementing it, liked the program, liked the experience). (2) *Comfort with topics and activities*: grade in which participants and/or facilitators liked topics and activities in the curriculum (e.g., comments about how much they liked the curriculum, what topics/activities they liked more or less). (3) *Understanding of content*: grade in which participants and/or facilitators understood (or have problems understanding) concepts and activities in the curriculum (e.g., comments about problems understanding concepts or activities instructions). (4) *Willingness to use the intervention in the future*: expressed willingness to continue implementing the intervention in schools.

**Quantitative measures from students’ post-test surveys.** (1) *Satisfaction*: students responded to four items on the post-test survey asking about how much they liked *Mantente REAL*, including its various components (videos, homework, classroom activities) and the program overall (0 = did not like it at all, 1 = did not like it much, 2 = liked it, 3 = liked it a lot). (2) *Comfort with topics and activities*: students reported in four post-test questions whether the program was interesting, fun, easy to pay attention to, or boring (0 = strongly disagree, 1 = disagree, 2 = agree, 3 = strongly agree).

#### Utility

The degree to which the program is perceived as useful in producing individual and social change (Murta et al., 2021) was explored through the following measures.

**Qualitative measures from teachers’ focus groups.** (1) *Knowledge*: information acquired and perception that contents are useful. (2) *Applicability*: similarity with participants’ life; authenticity of content. (3) *Impact*: changes in the lives of participants; changes in the work of facilitators and routine of services.

**Quantitative measures from students’ post-test surveys.** (1) *Knowledge*: **s**tudents reported on the post-test survey whether the program gave them useful information (0 = strongly disagree to 3 = strongly agree), and how much they learned from it (0 = not at all, 1 = a little, 2 = some, 3 = a lot). (2) *Applicability*: students assessed the authenticity of content in three post-test items, whether it was “like my life”, “like youths I know”, and like situations that they know other students get into. Two additional items assessed whether situations in the curriculum and characters in the video appeared “real” (0 = strongly disagree to 3 = strongly agree). (3) *Impact*: students reported whether they talked about the program with various people in their social network: parents, siblings, cousins, other family members, friends, and others. These were assessed separately (0 = no, 1 = yes) and as a count of the number of different categories of people they talked with (0 to 6).

### Data Analysis

Qualitative analysis was conducted following a thematic and framework approach to identify codes and analyze patterns (themes) among the teachers’ experiences with, and beliefs about, the *Mantente REAL* program and to effectively organize and interpret the data. Thematic analysis was conducting following Braun and Clarke’s (2006) recommendations: (a) read and become familiarized with the data, (b) generate initial codes in a systematic method, (c) identify themes based on available codes, (d) review codes and themes for accurate data representation, (e) define and name themes, and (f) select vivid examples to illustrate findings. Additionally, Gibbs (2018) and Rabiee (2004) recommendations on thematic coding and categorization of qualitative data (steps *b* to *d* of Braun & Clarke’s recommendations) were used as a reference during the data analysis process to increase consistency in coding and decrease misrepresentation of the data: e.g., creating codes that are analytical and theoretical and not merely descriptive; organizing codes hierarchically and comparing codes (which may be interpreted as a constant comparison analysis; Onwuegbuzie et al., 2009); and interpret data considering word meaning (*keywords-in-context* according to Onwuegbuzie et al., 2009), focus group context, and internal consistency throughout the focus group. To ensure that a consistent framework on social validity of prevention programs was reflected in the process of coding, members of the research team conducted a literature review to synthesize existing concepts for assessing social validity, which became the basis for the initial coding scheme (step *b* of Braun & Clarke’s recommendations). The codes were generated from keywords that define feasibility, acceptability, and utility after the agreement of three researchers based on the literature reviewed. Focus group data were transcribed verbatim in Spanish by the local team responsible for data collection in each site and two independent researchers utilized these Spanish-language transcripts to conduct the thematic analysis.

Quantitative analysis was conducted with SPSS 25 to analyze frequencies, other descriptive statistics (means, standard deviations, range), and *t*-tests to assess differences in teachers’ implementation and students’ perceptions of *Mantente REAL* between the two sites (i.e., Seville and Santiago de Compostela). Differences between sites on the evaluations of the program can be an indicator of the cultural appropriateness of the program after the cultural adaptation. Because it was adapted in Seville, the program might be viewed as more socially valid in that site.

## Results

### Qualitative results

A summary of qualitative results regarding the teachers’ opinions and experiences with the *Mantente REAL* program is displayed in Table 1.

**Table 1:** *Summary of Qualitative Results about the Teachers’ Opinions and Experiences with the Mantente REAL (MREAL) Program*

Themes	Codes	Findings
Feasibility	*Intervention fit*	Teachers perceived *MREAL* as well-designed to address substance use and peer pressure with adolescents beginning secondary school.
	*Implementation characteristics*	Teachers reported the manual facilitates delivery of *MREAL*. Some teachers reported challenges in managing large classroom sizes and implementing the program in the proposed timeframe. Overall, they would like to have more time to implement *MREAL*.
	*Practicality*	Teachers reported a high level of student participation and involvement with *MREAL*.
Acceptability	*Satisfaction*	Teachers reported high satisfaction with the experience of implementation and the curriculum of *MREAL*.
	*Comfort with curriculum*	Teachers noted that students enjoyed the *MREAL* activities and topics, especially the interactive, in-group activities and videos.
	*Understanding of content*	Teachers reported that students understood the activities in the *MREAL* curriculum, although with some difficulties when activities demanded a capacity for abstraction.
	*Willingness to use*	Teachers showed high willingness to continue using *MREAL* in their schools.
Utility	*Knowledge*	Teachers perceived their students gained knowledge from *MREAL*, especially the behavioral skills to resist drug offers.
	*Applicability*	Teachers viewed *MREAL* as highly applicable to daily life, representing real scenarios and potential situations that students might have to face. Teachers from Santiago de Compostela noted that *MREAL* would benefit from a greater cultural responsiveness in the Galician context.
	*Impact*	Teachers perceived changes in students due to *MREAL*, especially those related to the use resistance strategies. Teachers also reported changes in their own work, such as improving their skills for connecting with the students.

#### Feasibility

Teachers provided overall positive feedback about *Mantente REAL*, perceiving it to be a feasible program for implementation in Spain and to be a well-designed program to address substance use and peer pressure at the beginning of adolescence.

“*Es importantísima la prevención, en esta edad tan pequeña casi ningún programa* [se aplica] *. . para mí debería ser obligatorio*” (Teacher, Seville). “*Desde un principio nos pareció muy interesante este programa y creemos que es muy necesario empezar a tratar la presión de grupo en los primeros años de la secundaria*” (Teacher, Santiago de Compostela). [“Prevention is very important, at this very young age almost no program [is being implemented]. . for me it should be mandatory” (Teacher, Seville). “From the beginning we found this program very interesting and we believe that it is very necessary to start dealing with peer pressure in the early years of middle school” (Teacher, Santiago de Compostela).]

One of the characteristics of the program that teachers liked the most was the manualization of the intervention. It helped increase the teachers’ confidence in their ability to deliver effective program results, and reduce concerns that facilitators of *Mantente REAL* should have prior expertise in prevention. Teachers felt comfortable implementing the sessions due to the detailed guidelines presented in the manuals. This contributed to the high perceived practicality of the program, as teachers expressed their involvement in delivering the curriculum and noted positive student participation.

“*Estructurado, eso es fundamental, eso es lo que más me ha gustado el hecho que todo estuviera tan estructurado*”, “*Yo he estado a gusto y también he tenido respuesta por parte del alumnado*” (Teachers, Seville). “*Está todo muy explicado en los manuales y eso es una ayuda cuando no sabes del tema*”, “*Mostraron mucho interés en las tareas y actividades que se llevaron a cabo*” (Teachers, Santiago de Compostela). [“It is a structured program, that is fundamental, that is what I liked the most, the fact that everything was so structured”, “I was at ease and I have also had a response from the students” (Teachers, Seville). “Everything is well explained in the manuals and that is of great help when you do not know the subject”, “The students showed a lot of interest in the homework and activities that were carried out” (Teachers, Santiago de Compostela).]

Teachers mentioned some issues that affect feasibility, specifically regarding time concerns and the complexities of implementing the program given the certain constraints in the schools. For example, the 12-week long timeline (one lesson per week) or the large classroom size posed challenges. Teachers usually implemented the program during homeroom hours, so they had to manage the time to also address overlapping school activities and other activities inside and outside school. Therefore, teachers expressed some challenges completing all the activities in the curriculum on time and felt they needed more time to implement the program (or for the lessons to be shorter). They also mentioned that it would be better to implement the program in small groups. Teachers did not mention additional cost or resource implications (such as technical issues) of the program so the cost-effectiveness of the program was perceived as high.

“*Había temas en el aula que no podíamos tratar en la hora de tutoría, entonces estaban bien las 12 sesiones, pero a lo mejor cada 3 sesiones, un hueco*” (Teacher, Seville). “*Nosotros tenemos tutorías de casi 30 alumnos y se hace muy difícil manejar la clase con tantos niños*” (Teacher, Santiago de Compostela). [“There were topics in the classroom that we could not deal with at homeroom, so the 12 sessions were fine, but maybe we should have some extra time every 3 sessions” (Teacher, Seville). “We have classrooms of almost 30 students and it becomes very difficult to manage the class with so many children” (Teacher, Santiago de Compostela).]

#### Acceptability

On the other hand, teachers confirmed the acceptability of *Mantente REAL* as a prevention program for early adolescents in Spain. Teachers expressed their satisfaction with the implementation experience and with the curriculum of the program.

“*El programa en sí está fantástico*” (Teacher, Seville). “*La experiencia fue muy buena*” (Teacher, Santiago de Compostela). [“The program itself is fantastic” (Teacher, Seville). “The experience was very good” (Teacher, Santiago de Compostela).]

Teachers noted the students liked the activities very much, especially the interactive activities and the videos. Although some teachers pointed out that early adolescents may have difficulty participating in activities that require greater capacity for abstraction (such as contrasting personal with family values), teachers generally reported a high degree of comfort with topics and activities included in the curriculum, for themselves and their students.

“*Para mí son los dos grandes éxitos: prácticas y vídeos*” (Teacher, Seville). “[Los estudiantes] *Prefieren hablar y compartir experiencias de forma dinámica. Creemos que es un material muy bueno*”, “*Los vídeos, por ejemplo, les encantaron*” (Teachers, Santiago de Compostela). [“For me the role-playings and the videos are the two great successes of the program” (Teacher, Seville). “[Students] prefer to talk and share experiences in group. We think it’s very good material”, “They loved the videos, for example” (Teachers, Santiago de Compostela).]

Ultimately, teachers reported high willingness to use the intervention in the future with new students starting middle school, but also in later grades as they thought the topics and activities of *Mantente REAL* are acceptable for use with older students and can serve as a reinforcement of previous learning.

“*Las cuatro habilidades están geniales y no solo para este curso, me gustaría seguir usándolas*” (Teacher, Seville). [“The four strategies are great and not only for this course, I would like to continue using them” (Teacher, Seville).]

#### Utility

Teachers viewed *Mantente REAL* as a highly applicable prevention program for Spanish early adolescents. They mentioned specifically the utility of learning behavioral skills (the REAL strategies) to deal with substance use situations. The REAL strategies were also considered useful while dealing with other risky situations.

“*Las estrategias que han aprendido luego le servirán para el consumo y para otra cosa*” (Teacher, Seville). “*Mantente REAL es una forma interactiva de aprender ciertas habilidades que pueden ser útiles a los niños más allá del consumo de drogas*” (Teacher, Santiago de Compostela). [“The strategies they have learned will serve them later for [substance] use, and for other things” (Teacher, Seville). “*Mantente REAL* is an interactive way to learn certain skills that can be useful to children beyond drug use” (Teacher, Santiago de Compostela).]

Teachers from both sites commented that materials overall were applicable to the students’ daily life, although teachers from Seville sometimes perceived the videos, language and the situations as more similar to their students’ lives because the materials were developed and adapted in their city.

“*La cosa buena* [de los vídeos] *es que, al estar rodado aquí en Sevilla por chicos de aquí, le sonaban muy cercanos*” (Teacher, Seville). “*Comentaron en más de una ocasión que les gustaría que reflejasen más el contexto gallego. Muchas veces no se entendía lo que decían*” (Teacher, Santiago de Compostela). [“The good thing [about the videos] is that, being shot here in Seville by youth from here, they sounded very close to the students’ [way of speaking]” (Teacher, Seville). “They commented on more than one occasion that they would like them to reflect more of the Galician context. Many times, they did not understand what they were saying [in the videos]” (Teacher, Santiago de Compostela).]

The impact of the knowledge acquired, that is, the changes in students’ lives because of the program implementation, was reported more specifically in connection to the resistance strategies. Teachers themselves also expressed that *Mantente REAL* impacted how they work at school.

“[Los estudiantes] *Saben perfectamente lo que es REAL*”, “*Es un programa que me ha ayudado mucho, a trabajar, a conectar con los niños, a presentarles herramientas*” (Teachers, Seville). [“[The students] know perfectly what REAL is”, “It is a program that has helped me a lot, to work, to connect with the children, to present them with tools” (Teachers, Seville).]

### Quantitative results

#### Quantitative results from student’s post-test surveys

Students reported very positive evaluations of the *Mantente REAL* curriculum (see Table 2). As an indicator of program feasibility, students were highly engaged with the material as measured by the level of student participation (78% reporting participating some or a lot) (“practicality”). As an indicator of acceptability (“satisfaction”), more than half of the students agreed that they were satisfied with the program (over 80% said that overall, they liked or liked the program very much), as well as its components, especially the videos (about 85% liked or very much liked the videos). Over 75% of students rated the program as acceptable (interesting and easy to pay attention), as well as fun (66%) and not boring (73%), which are indicators of acceptability regarding “comfort with topics and activities” and “understanding of content”. As for utility, in assessments of knowledge gained, over 75% said the program gave “some” or “a lot” of useful information, about 65% said they learned “a lot”, and about another 20% said they learned “some” (“knowledge”). About 75% of the students agreed that the program was credible and that the characters in the videos seemed real (“applicability”), while some of them felt that the situations in the curriculum and videos were like their lives (about 20%) or applicable to youths they knew (about 35%). One indicator of the “impact” of the program was that more than half of the students (about 60%) talked about it with others, mostly with their parents and/or with friends.

**Table 2:** *Descriptive results from Student’s Post-Test Surveys: Frequencies on Evaluations of the Mantente REAL Curriculum (n = 312)*

	Not at all (%)	A little (%)	Some (%)	A lot (%)
**FEASIBILITY**				
Practicality:				
How much did you participate in the program?	6.5	15.2	37.9	40.5
**ACCEPTABILITY**				
Satisfaction:				
Liked the program overall	5.8	14.1	46.5	33.7
Liked the videos	3.9	10.0	36.0	50.2
Liked the homework	12.9	21.2	36.7	29.3
Likes the classroom activities	8.0	14.5	40.8	36.7
Comfort:				
The program was interesting	7.7	12.9	32.6	46.8
It was fun	14.0	20.1	33.1	32.8
It was easy to pay attention to	8.1	13.3	30.1	48.5
The lessons bored me	40.0	33.2	13.5	13.2
**UTILITY**				
Knowledge:				
The program gave me useful information	11.4	12.0	21.8	54.9
How much did you learn from the program?	7.5	8.8	19.2	64.5
Applicability:				
The program was credible	8.5	13.0	18.9	59.6
It was like my life	63.8	17.4	10.5	8.2
It was like youths I know	45.1	19.5	17.5	17.9
I know youth who get into situations like these	39.2	24.3	14.6	22.0
The video characters seemed real	12.0	13.9	18.1	56.0
Impact:	No (%)	Yes (%)		
Talked about program: parents	38.8	61.2		
Talked about program: siblings	75.8	24.2		
Talked about program: cousins	88.4	11.6		
Talked about program: other family members	72.1	27.9		
Talked about program: friends	36.1	63.9		
Talked about program: others	84.7	15.3		

*Note*. For simplicity, the responses were unified under four common categories. The specific terms used in the range responses to each item was described in the Measurements subsection

On most indicators, students from Seville reported more positive evaluations of *Mantente REAL* than the students from Santiago de Compostela (see Table 3). In *t*-tests, most of the mean differences were statistically significant, including on measures of practicality, satisfaction, comfort, knowledge and applicability (every category except impact). There were no differences by city in measures such as level of participation in the curriculum activities, how much students liked the program overall, whether it was boring, whether the program was like their lives, whether they knew youth who get into situations like those presented in the videos, and whether they talked to others about the program.

**Table 3:** *Results from Student’s Post-Test Surveys: Differences between Sites on Evaluations of the Mantente REAL Curriculum (n = 312)*

	Seville		Santiago		*t*	Range
	*M*	*SD*	*M*	*SD*		
**FEASIBILITY**						
Practicality:						
Number of program videos viewed	4.06	1.02	4.33	0.88	-2.36^*^	0–5
How much did you participate in the program?	2.14	0.83	2.18	0.93	-0.37	0–3
**ACCEPTABILITY**						
Satisfaction:						
Liked the program overall	1.87	0.82	1.86	0.94	0.15	0–3
Liked the videos	2.46	0.65	2.26	0.87	2.24^*^	0–3
Liked the homework	2.08	0.86	1.62	1.07	4.06^***^	0–3
Likes the classroom activities	2.24	0.79	1.91	0.98	3.20^**^	0–3
Comfort:						
The program was interesting	2.39	0.77	2.04	1.02	3.40^**^	0–3
It was fun	2.14	0.84	1.64	1.12	4.32^***^	0–3
It was easy to pay attention to	2.40	0.81	2.01	1.02	3.66^***^	0–3
The lessons bored me	0.88	1.00	1.10	1.06	-1.81^†^	0–3
**UTILITY**						
Knowledge:						
The program gave me useful information	2.40	0.87	2.08	1.12	2.73^**^	0–3
How much did you learn from the program?	2.64	0.72	2.25	1.02	3.78^***^	0–3
Applicability:						
The program was credible	2.53	0.81	2.13	1.10	3.53^***^	0–3
It was like my life	0.68	0.95	0.62	1.00	0.57	0–3
It was like youths I know	1.25	1.14	0.93	1.15	2.42^*^	0–3
I know youth who get into situations like these	1.32	1.16	1.06	1.16	1.86^†^	0–3
The video characters seemed real	2.38	0.93	2.03	1.15	2.86^**^	0–3
Impact:						
Talked about program: parents	0.64	0.48	0.61	0.49	0.46	0–1
Talked about program: siblings	0.25	0.44	0.24	0.43	0.20	0–1
Talked about program: cousins	0.12	0.32	0.12	0.32	0.00	0–1
Talked about program: other family members	0.29	0.46	0.28	0.45	0.11	0–1
Talked about program: friends	0.65	0.48	0.65	0.48	-0.08	0–1
Talked about program: others	0.16	0.37	0.16	0.37	0.12	0–1
# of different people talked to about program	2.07	1.59	2.03	1.61	0.25	0–6

^***^p < .001. ^**^p < .01. ^*^p < .05. ^†^*p* < .10

#### Quantitative results from researchers’ fidelity observation forms

Table 4 shows the closed-ended ratings from the observation forms that provide information about program feasibility, separately for the two cities: teacher fidelity to the manualized curricula and practicality as indicated by student participation. On average, teachers from both sites completed 8 or more of the 10 activities on the fidelity checklists. As indicators of fidelity, teachers from both sites “mostly” or “completely” followed the lesson plans, were prepared to facilitate the lesson and were informed about the content, gave clear instructions, and motivated students’ participation. Teacher classroom management was rated as “good” or “excellent,” the pace of instruction was right, and only a few teachers in Santiago de Compostela inserted content not included in the manual (with significant mean differences between groups). The similarly high ratings of fidelity in both sites limited statistically significant differences to two other measures: teachers in Seville were rated as better prepared to deliver the lesson and gave clearer instructions. Observers also noted similarly high levels of student participation and attention to the videos in both sites, indicators of practicality.

**Table 4:** *Results from Researchers’ Fidelity Observation Forms (N = 6)*

	Seville		Santiago		*t*	Range
	*M*	*SD*	*M*	*SD*		
**FEASIBILITY**						
Fidelity:						
Completed checklist items	9.11	1.13	8.46	1.53	1.52	0–10
Followed the lesson plan	3.67	0.59	3.38	0.71	1.41	1–4
Was prepared to deliver the lesson	3.83	0.38	3.50	0.59	2.21^*^	1–4
Well informed about program content	3.67	0.49	3.50	0.66	0.90	1–4
Gave clear instructions	3.83	0.38	3.42	0.65	2.59^*^	1–4
Motivated student participation	3.67	0.59	3.63	0.65	0.21	1–4
Managed the group process well	2.44	0.62	2.46	0.59	-0.07	1–3
Appropriate pace (not slow or fast)	2.61	0.78	2.58	0.78	0.12	1–3
Added content not in the manual	0.00	0.00	0.17	0.39	-2.15^*^	0–1
Practicality:						
Student participation	2.61	0.50	2.42	0.58	1.13	1–3
Students appeared engaged	3.94	0.64	3.71	0.86	0.98	1–5
Students attentive to the videos	3.64	0.50	3.69	0.60	-0.22	1–4

^*^p < .05.

## Discussion

The objective of this study was to analyze the feasibility, acceptability, and utility of the *Mantente REAL* program in the first grade of Secondary Compulsory Education in Spain. *Mantente REAL* is a culturally adapted program that showed preliminary evidence of efficacy in preventing drug use in a small RCT study (Cutrín et al., 2020). The current study highlights the importance of evaluating not only the efficacy and effectiveness of a prevention program, but also its social validity, determining its quality and social impact. The findings overall captured positive perceptions of teachers, students, and observer researchers about the *Mantente REAL* program. They viewed it as a feasible, acceptable, and useful program to prevent substance use in early adolescents in Spain. Considering culturally appropriateness, differences between sites indicated that the program is perceived more positively by those participants living in the specific cultural context in which the cultural adaptation of the curriculum was conducted (i.e., Seville).

The high level of substance use among European and Spanish populations, and young adolescents in particular, highlights the need for interventions focused on early universal prevention. Establishing the scientific and social validity of interventions is needed in order to change actual substance using behaviors by teenagers. These findings support the premise that a reduction in risky behaviors among adolescents can happen by implementing programs that are not only evidence-based (Delegación del Gobierno para el Plan Nacional sobre Drogas, 2019) and culturally adapted (Holleran et al., 2002), but that are also socially validated.

Following this line of argument, it is essential to consider the degree of effective application and the recognition of the different actors involved in the intervention. In this study, social validity was measured with the levels of feasibility, acceptability, and utility by the two main stakeholders in the intervention process: teachers and students, as well as observer field researchers. Information provided by the actors involved in the intervention for this study is consistent with the widely affirmed view that participation in the entire process of the design, cultural adaptation, implementation and evaluation of prevention interventions increases their social validity (Gosin et al., 2003).

One of the outstanding processes that guarantee the success of a prevention program through the inclusion of the culture of the target population is the co-design of the content of the intervention materials, as shown by the higher positive feedback provided by students in Seville, the city in which the materials and videos were adapted and recorded. Culturally tailored programs are based on the premise that including youth culture in the content and format of the prevention message will reduce adolescent drug use (Kandel, 1995; Holleran et al., 2002). Indeed, the findings support the concept that adaptation models based on high level of community involvement and participation produce strong prevention interventions (Castro et al., 2004, 2010; Wingood & DiClemente, 2008).

In our study, elements that stand out as contributing to the feasibility of the program include key strategies to consistently protect the core elements of the curriculum while adapting and transmitting culturally adapted messages in the most effective way. Included among these are the detailed manualization of the curriculum and the extensive training of regular classroom teachers (Harthun et al., 2009). The training of teachers and the manualization of the program offer a valuable opportunity to reproduce its contents faithfully and implement the program with fidelity.

This study, in comparison to others (e.g., Murta et al., 2021), additionally included the external perspective of researchers by completing standardized observation forms. Other studies collected field notes from researchers (Lohan et al., 2018), but did not present specific findings in this regard. Researchers’ views on feasibility (fidelity and practicality-students’ participation) help validate the personal perspective of teachers/facilitators and students and provide another valuable resource of information to assess program implementation.

On the other hand, teachers identified challenges to feasibility, such as the need to adjust to school structural constraints, such as their suggestions to increase the number of sessions and/or their duration, and offer the curriculum in smaller groups of students. The issue of class size may relate back to the original design of the curriculum for middle school classrooms in the United States that often have 25 or fewer students. With larger class sizes, alternatives might be needed when implementing highly interactive classroom activities like role-playings.

A key element of program acceptability was satisfaction with its content and format. Teachers and students strongly endorsed *Mantente REAL*’s highly practical and participatory activities and videos, in line with the view that programs based on participatory learning are more widely accepted (Gosin et al., 2003). The combination of oral, written, and audiovisual supporting materials facilitated acceptability of content and activities and it increased the potential effectiveness of the program (Espada et al., 2015).

The ability of a program to promote individual and social changes in daily life is a key element of program utility. *Mantente REAL* impacted students’ lives, not only in restraining consumption of alcohol and other drugs, but more generally by imparting skills for navigating life challenges (Cutrín et al., 2021). Students and teachers recognized that these skills were made accessible through effective curriculum and video messaging, and the participatory teaching and learning style. From the eco-evolutive perspective (Marsiglia & Holleran, 1999), a contextualized preventive intervention that develops assertive responses and resilience in adolescents in their own relational environments is essential for improving the learning of social and behavioral skills. In this regard, a key contribution to the social validity of *Mantente REAL* was that regular teachers were able to implement the intervention with fidelity, rooted in the established social contexts of their school classrooms (Horner et al., 2005).

Finally, it is relevant to note that around 60% of students reported “it was not at all like my life” and around 45% reported “it was not at all like youths I know”. Considering the other positive indicators of feasibility, acceptability, and utility, these findings might be an indicator of early adolescence as the ideal age to prevent risky situations, as previous research indicated (Gottfredson & Wilson, 2003). Developmentally, many students did not feel the specific situations implicating substance use offers and consumption were situations that they had yet experienced. Therefore, anticipating these situations, teaching resistance strategies to substance offers, and promoting socioemotional skills to face risky situations, can effectively prevent later adolescent involvement in substance use and/or other unhealthy behaviors (Sancassiani et al., 2015).

## Limitations

The study’s assessments of the social validity of *Mantente REAL* were limited by aspects of the research design, available data sources, and challenges in measuring complex concepts. First, the results reflect the views of teachers and students from a convenience sample of schools from two cities in different regions of Spain. There is a need for future optimization research that examines the social validity of *Mantente REAL* in different parts of Spain. Second, available information on social validity was restricted to short-term assessments, which make it difficult to establish whether effects relating to the utility and effectiveness of the program will persist over time. Third, conceptually complex aspects of social validity, such as acceptability and applicability, are difficult to gauge reliably and may have wide variations across subpopulations and different social settings. For example, differing depending on social dynamics in particular classrooms and with different teacher-facilitators. Finally, results showing differences between the two cities in social validity assessments suggest that reflections of particular social contexts in the cultural program adaptation –such as the setting of the videos in Seville and the use of language identified with a particular region– may color these assessments.

## Conclusions

The high prevalence of substance use and abuse in Spain, among youths and adults, highlights the need to disseminate effective prevention approaches and conduct systematic research to improve the quality, reach, and social validity of prevention programs. Teachers and students provided strong and notably consistent evidence that the culturally adapted version of *Mantente REAL* for Spain (shown as efficacious in a small RCT) was viewed as: feasible for implementation, although with challenges related to school structural constraints; acceptable in terms of satisfying and comfortable content; and of utility in prompting desired individual change.

Based on these findings demonstrating the social validity of *Mantente REAL*, we recommend that certain elements need to be incorporated into the design and testing of prevention programs: (1) systematic data collection to establish an evidence base, (2) rigorous cultural adaptation through a process of co-design with the different actors involved in the intervention, (3) an understanding of the context and cultural specificity on the part of the program facilitators, (4) appropriate training of the facilitators to deliver the program with fidelity, and (5) engage the active participation of the target population. The adaptation, implementation and testing of *Mantente REAL* in Spain followed these steps, which produced a very promising end result.
